# Primary Care Patient and Clinician Perspectives on Safer Use Strategies for Opioids and/or Stimulants: A Mixed-Method Study

**DOI:** 10.1007/s11606-025-09418-5

**Published:** 2025-03-04

**Authors:** Brittany E. Blanchard, Elizabeth J. Austin, Erin Chase, Julien Rouvere, Vinita Sharma, Morgan Johnson, Nichole Sams, Florence Williams, Madeline C. Frost, Sarah Leyde, Judith I. Tsui, Susan E. Collins, John C. Fortney

**Affiliations:** 1 Department of Psychiatry and Behavioral Sciences, University of Washington School of Medicine, Seattle, WA USA; 2 Department of Health Systems and Population Health, School of Public Health, University of Washington, Seattle, WA USA; 3 Richard M. Fairbanks School of Public Health, Indiana University, Indianapolis, IN USA; 4 Department of Human-Centered Design and Engineering, University of Washington, Seattle, WA USA; 5 Veterans Affairs (VA) Health Systems Research, Center of Innovation for Veteran-Centered and Value-Driven Care, VA Puget Sound Health Care System, Seattle, WA USA; 6 Department of Medicine, School of Medicine, University of Washington, Seattle, WA USA

**Keywords:** harm reduction, opioids, stimulants, primary care, protective behavioral strategies

## Abstract

**Introduction:**

Safer use strategies (SUS) are behaviors before, during, and after drug use to moderate use and/or mitigate unwanted consequences. As treatment of substance use disorders becomes more common in primary care, offering SUS in primary care merits exploration.

**Method:**

We explored acceptability and use of SUS in primary care using a convergent parallel mixed-method design consisting of patient and clinician semi-structured interviews and surveys. Participants were recruited from primary care clinics involved in a multi-state practice research network. Patients with lifetime stimulant and/or opioid and any SUS use were eligible. All clinicians were eligible. Qualitative data were analyzed using a rapid assessment procedure. Quantitative data were analyzed descriptively.

**Results:**

Participants included patients (*n* = 10) and clinicians (*n* = 12) from multiple disciplines. More than half of patients indicated that every SUS surveyed should be offered in primary care. Patients reported using multiple SUS to stay safer, reduce consequences, and limit use. Clinicians reported that offering SUS to primary care patients is acceptable and supported SUS use by sharing informational resources (e.g., safer injection practices) and tangible resources (e.g., naloxone, medication for opioid use disorder [MOUD]). Some strategies recommended by patients were not currently being systematically offered (e.g., fentanyl test strips). Several clinicians expressed willingness to discuss SUS with patients but wanted more training and resources to facilitate SUS discussions to support patient goals.

**Conclusion:**

Offering SUS to primary care patients is acceptable to patients and clinicians. Clinicians supported some SUS use, though more SUS and harm reduction training and resources were desired. Providing SUS to patients who use stimulants and/or opioids could enhance patient-centered primary care, especially in clinics offering MOUD. More research is needed to optimize SUS support in primary care settings.

**Supplementary Information:**

The online version contains supplementary material available at 10.1007/s11606-025-09418-5.

## INTRODUCTION

Drug toxicity is a leading cause of injury-related death in the United States (U.S.),^1^ with more than 110,000 deaths in 2022.^2^ Most deaths involved illicitly manufactured fentanyl, other opioids, and stimulants.^1–3^ Unsafe drug use contributes to numerous medical sequelae. Some are specific to drug class (e.g., stimulant-induced psychosis) or administration route (e.g., injection-related infections, sepsis),^4,5^ but many negative outcomes are not unique to one class or route (e.g., viral hepatitis, pulmonary edema, sexually transmitted infections).^6–9^ Drug-related medical sequalae and deaths are preventable, but access to harm reduction programs and substance use disorder treatment remains limited and inequitable.^10,11^

Primary care (PC) can be more accessible and less stigmatizing than addiction specialty care and is often the first point of contact within the healthcare system.^12^ Recent efforts have increased access to medication for opioid use disorder (MOUD) through PC;^13^ however, in 2022, fewer than 40% of people with OUD diagnoses received MOUD. Further, no FDA-approved medications currently exist for the 4.5 million people with stimulant use disorders (StUD)^9,14^ though some medications are prescribed off-label.^15^

Safer use strategies (SUS), also called harm reduction strategies or protective behavioral strategies,^16–18^ are ways to stay safer and healthier while using.^19,20^ SUS from grassroots harm reduction movements,^21,22^ public health, and syringe service programs indicate SUS like overdose education and carrying naloxone, not using alone, and safer injection practices reduce overdose death and infectious disease transmission, respectively.^23,24^ SUS improve outcomes, and PC may be an appropriate clinical setting to increase population-level access to SUS education and resources.

A systematic review found that prescribing naloxone is acceptable and feasible in PC.^25^ Current best practices in PC include discussing safer injection behaviors.^5^ However, smoking has replaced injecting as the most common route used in drug-related deaths,^26^ so identifying acceptable SUS for PC patients across routes of administration is critical and time-sensitive.

The goals of this research were to explore perceptions of acceptability of offering SUS to patients in PC settings and characterize SUS used by PC patients who use stimulants and/or opioids and ways PC clinicians currently support patient SUS use. We also elicited suggestions/recommendations to improve SUS support. If acceptable, offering SUS in PC could increase access to patient-centered, harm reduction-oriented care.

## METHOD

### Procedures

#### Study Overview and Participant Recruitment

We used a complementary parallel mixed-method design.^27^ We used qualitative interviews to identify SUS most salient to patients, SUS supported by clinicians, and perceptions around specific strategies for PC. We used survey data to quantify patient SUS use and perceived acceptability. We recruited two clinics in urban Washington State and one clinic in Montana from the Washington, Wyoming, Alaska, Montana, and Idaho (WWAMI) region Practice and Research Network (WPRN), a PC practice-based research network of clinics and clinical organizations. Clinicians were recruited from all three clinics, and patients were recruited from the participating Washington clinics via electronic health record query followed by opt-out letters or recruited by on-site clinicians.

Patients were eligible if they reported any lifetime opioid and/or stimulant use, any SUS use, access to phone/internet for 1 hour, and spoke English. Most patient participants were recruited via purposive sampling stratified by opioid/stimulant-related codes, gender, and race from an electronic health record query followed by opt-out letters. One clinic recruited by on-site clinicians (*n* = 1). Patients were screened over the phone, and eligible patients were played an audio recording of the consent form over the phone by a research team member who answered questions. Clinicians at participating clinics were eligible. Clinicians were e-mailed an information sheet containing study-related details by the clinical champion and could ask questions via email or prior to the interview. Verbal consent was documented via interview recording for all participants.

##### **Data Collection**

Patient participants completed a 40-min semi-structured interview (see Appendix 1), followed by a 20-min survey about SUS (33 strategies compiled and adapted from multiple resources^28–31^; see Table 2 for list), drug use experience (modified items from the Addiction Severity Index^32^), and demographics. Patient participants were provided a copy of the consent form via email or mail based on their preference. All patients chose to complete the study via telephone.

Clinicians completed an hour-long interview via HIPAA-compliant interactive video software (see Appendix 2) and sent a 10-min online survey via REDCap^33,34^ which included a question rating the acceptability of “increasing provision of harm reduction strategies at their clinic” (0=*extremely unacceptable*, 5=*extremely acceptable*) and demographics. One or two research team members conducted patient interviews, and the principal investigator (PI) led all clinician interviews with one to two research team members taking notes. Participants were compensated with gift cards ($50 and $100 for patients and clinicians, respectively). We conducted interviews until theme-level saturation was reached. All procedures and materials were approved by the University of Washington Institutional Review Board.

##### **Data Analysis**

The research team transcribed patient interviews, and we used interactive video software transcription for clinician interviews. The PI reviewed and cleaned all transcripts. The PI and two to three research team members independently coded transcripts using a deductive approach. The team reviewed codes and resolved discrepancies by transcript review and discussion. We conducted iterative team reviews weekly throughout coding to review codes, identify emergent codes, and reach consensus.

We coded SUS into three a priori themes based on a framework developed by the Harm Reduction Research and Treatment (HaRRT) Center through community-based participatory research.^35–37^ This framework was created with people with lived/living use experience^36^ and includes strategies for patients at any stage of change.^38^ Acceptability and subthemes within each SUS category were derived based on the research questions. We analyzed quantitative survey data descriptively, and we integrated qualitative and quantitative data using joint displays to enhance interpretation.^39^

## RESULTS

### Participant Sample Description

The study sample included 22 individuals (10 patients and 12 clinicians) from clinics with established integrated behavioral health and MOUD programs. Participant demographic descriptive statistics are in Table 1. See Tables 3 and 4 in the Appendix for additional patient demographics, drug experience(s), and route(s) of administration used. Clinician participants included attending physicians, four resident physicians, one registered nurse practitioner, one nurse, and one pharmacist (average experience = 8.60 years).

**Table 1:** Patient and Clinician Participant Demographics

Demographics	Patients (*n* = 10)*	Clinicians (*n* = 12)
Age mean (SD)	53.00 (14.40)	36.27 (9.26)
Range	(35–75 years)	(26–53 years)
Race and Ethnicity^†^ *n* (%)		
Asian/Asian American	1 (10.00%)	2 (16.67%)
Black/African American	5 (50.00%)	0 (0.00%)
Latina/o/e	1 (10.00%)	0 (0.00%)
Native American/Alaska Native	1 (10.00%)	1 (8.33%)
White	5 (50.00%)	9 (75.00%)
Prefer not to answer/missing	2 (20.00%)	1 (8.33%)
Gender identity *n* (%)		
Man	5 (56.00%)	3 (25.00%)
Woman	4 (44.00%)	8 (66.67%)
Prefer not to answer/missing	1 (10.00%)	1 (8.33%)
Sexual orientation *n* (%)		
Bisexual	1 (10.00%)	0 (0.00%)
Gay/lesbian	1 (10.00%)	0 (0.00%)
Straight	6 (60.00%)	11 (91.7%)
Prefer not to respond/missing	2 (20.00%)	1 (8.33%)

*Note*. No participants identified as trans or non-binary

^*^n=1 missing for all patient demographic variables

^†^Participants could select multiple racial and ethnic identities. Percentage will not total to 100%

## Themes

First, we present data on the theme acceptability of offering SUS in PC. SUS data are then presented, organized into the three themes: (1) ways to be safer and healthier without changing use, (2) ways to use more safely, and (3) ways to change how much you use. Within each are the following subthemes: (a) patient use, (b) clinician supports, and (c) suggestions/recommendations. Last, we present suggestions/recommendations that span SUS categories.

### Acceptability of Offering SUS in Primary Care

Patients and clinicians found offering SUS in PC to be an acceptable practice. More than half of patients recommended all 33 SUS be offered in PC, and 100% of patients recommended 10 of the strategies (Table 2). Clinicians rated increasing provision of harm reduction strategies as “acceptable” (42%) or “extremely acceptable” (58%) in surveys. Clinicians used strategies to support SUS use across all themes and expressed a desire to learn more SUS to better support patients during interviews. Most patients also expressed high enthusiasm for offering SUS to patients in PC, as summarized by one patient, “For a lot of people, primary care is a lot of where the issue started, with the overprescribing […] For primary care doctors to use a harm reduction model in some way, I think that sounds good. I think it’s positive for sure” (Patient 6).

**Table 2:** Number of Patients Reporting SUS Use and Recommendations for Strategies that Should be Offered in PC

Safer use strategies	Strategies used by patients *n* = 10	Strategies that should be offered in PC *n* = 9
**Ways to be safer and healthier without changing use**		
Know the signs of overdose	6	9
HIV/hepatitis C/hepatitis B screening	9	9
Take care of your mouth	4	9
Take care of your mental health	7	9
Wound care	2	9
Take care of your veins (if you inject)	3	8
Let your body rest between uses	5	8
Seek out/use social support	6	8
Carry rescue drugs (Narcan, naloxone)	4	8
Prepare for safer sex (condoms)	4	8
Stay hydrated while using	6	7
Use medications as prescribed by physician	5	7
Avoid using alone	5	7
Test your drugs (use fentanyl test strips)	1	7
Eat before/during/after use (healthy food if possible)	7	6
Spotting (telling someone when you plan to use)	4	6
**Ways to use more safely**		
Choose safer ways to use	8	9
Avoid mixing opioids with stimulants (“speedballing”)	3	9
Shoot safer	5	9
Use in a safe place	10	8
Avoid mixing with alcohol	6	8
Smoke safer	4	8
Do not drive while using/under the influence	8	8
Use with safe people	8	7
Avoid mixing depressants (opioids and benzos like Xanax)	6	7
Restrict access to guns while using/under the influence	4	7
Only buy drugs from a trusted source	7	6
**Ways to change how much you use **		
Talk to a provider about medication/treatment/resources	9	9
Talking to a provider about withdrawal	9	9
Use less at a time	8	8
Choose not to use (take tolerance breaks)	4	8
Buy less at a time	5	7
Only use after completing the day’s responsibilities	7	6

*Note.* One patient participant discontinued the survey prior to completing questions reported in final column

### Ways to be Safer and Healthier Without Changing Use

#### Patient Use

SUSs that buffered the effects of stimulants and/or opioids while not requiring changing use included strategies that reduce drug-related consequences for themselves and others, supported physical and mental health, and addressed drug-related medical conditions. Patients with current living experience commonly reported having naloxone available as a strategy. Although patients reported adequate access to naloxone in interviews, they acknowledged that naloxone alone was inadequate: “Well, I have Narcan here at home, but I live by myself. And the thing about Narcan […] if you tend to OD, you can’t use the spray to bring you back” (Patient 6).

Some patients stated that preventing overdose among peers in the context of fentanyl contamination was a top priority, and strategies included carrying naloxone, checking others when “nodding” (i.e., unintentionally drifting in and out of consciousness, a common effect of opioids), overdose education, and sharing knowledge on contaminants. One patient who lost friends to fentanyl overdose said, “I feel everybody needs to know […] I’ve been telling my friends and family, “Look, you’re not just smoking your drug of choice […] we have been given something that we never asked for […] fentanyl” (Patient 2).

Patients reported using strategies like infectious disease screening/treatment, resting between use, vein care, seeking wound care, oral hygiene, and taking care of their body to support their physical health in surveys. Patients frequently endorsed eating around use in surveys and interviews. Patients expressed that support from friends, family, and group therapy were critical during interviews and commonly endorsed social support in surveys. Patients reported coping with trauma and mental health symptoms as a common reason for drug use and endorsed mental health care in surveys. Many patients reported experiencing trauma and disclosed mental health conditions (e.g., anxiety, depression, posttraumatic stress disorder, bipolar disorder). They reported psychotherapy and pharmacotherapy as two helpful SUS.

#### Clinician Supports

Clinicians supported many SUS commonly endorsed by patients. All clinics offered infectious disease screening/treatment and had integrated care models. One patient shared his PC team persistently recommended (but did not require) mental health treatment while he received MOUD, which led to him receiving and benefiting from psychotherapy. Clinicians also offered naloxone for patients and others in their lives. As shared by one clinician, “I have one patient, like he says he never needs [naloxone]. He smokes fentanyl all the time, but he said he saved a friend’s life with Narcan, so he’ll take some” (Clinician 1).

Naloxone distribution varied by clinic (prescription v. available onsite). One clinician whose clinic provided free distribution on site offered naloxone in paper bags to protect patient privacy, which was deemed especially important in rural communities where drug use and seeking any treatment are highly stigmatized.

Like patients, clinicians also provided education on contaminants, especially fentanyl:

I’m not sure that people here quite understand the risk for overdose and dying on fentanyl, so that’s the conversation that I have. That’s what I tell them that I lose sleep about […] ‘Yeah, sure you’re stable. You’ve been on Suboxone for 3 months, but you run out, and you […] need a refill, and you go out and get one fentanyl pill, and I mean that’s it’ (Clinician 3).

Although no patients discussed xylazine (i.e., an animal tranquilizer), clinicians who previously worked on the east coast expressed concerns regarding severity of wounds and depressant effects of xylazine combined with fentanyl.

Clinicians noted injection use-related wounds were a common presenting problem across stimulants and opioids. All three clinics offered wound care. One clinic offered wound care kits to patients with instructions for monitoring infections.

Few clinicians described counseling patients on timing meals around use and drinking water. One clinician prescribed medication for patients using stimulants to facilitate eating: “I have given some patients [who are using methamphetamine] Zofran for severe nausea that they are getting that stops them from being able to eat” (Clinician 7).

Another clinician offered oral hygiene kits (e.g., toothpaste, toothbrush) to patients and refers them to a co-located dental clinic to reduce access barriers.

Some clinicians reported exploring underlying conditions and reasons for use (e.g., sleep difficulties, reducing fatigue) and providing treatment for those problems. Most clinicians (and some patients) cited physical pain as a common presenting problem, as noted by one clinician: “Any exacerbation of pain, I find patients will just backslide pretty rapidly, so anything to help manage pain control, whether it’s [..] small bottles of Tylenol or ibuprofen, or anything that [..] helps take the edge off in a way” (Clinician 7).

#### Suggestions/Recommendations

One clinic had a state-funded grant to distribute naloxone without a prescription, and clinicians in other clinics indicated this would reduce naloxone access barriers for patients. Most patients (67%) indicated they would take fentanyl test strips for themselves and/or friends if offered in PC, as shared by one patient:

“I’ve had two friends actually die because they were given what they thought was crack cocaine, but it actually was fentanyl […] and that could have been prevented had they used like, a test strip to test the drugs […] having something like that [in primary care] is definitely, definitely a plus” (Patient 7).

However, neither patients nor clinicians knew where to access strips locally.

In addition to offering fentanyl test strips (state laws permitting), clinicians recommended offering naloxone for a friend, more training on SUS, patient- and clinician-facing SUS resources to facilitate SUS discussions, standardized naloxone distribution to all patients who use opioids (not just those on MOUD or prescribed opioids), and updated information about current drug contaminants in the community. Other clinician suggestions included providing resources to increase social support, as well as tangible resources, such as socks, nutrition bars, and protein shakes. As summarized by one clinician: “I would love to have the physical, tangible things I’m telling them they need to be doing to be safer […] food, water, hygiene, wound care […] and then, of course, access to all of the resources” (Clinician 7).

### Ways to Use More Safely

#### Patient Use

Patients reported using in a safe place with trusted people, safer injection/smoking practices, safer routes of administration, not mixing certain drugs, and buying drugs from trusted sources in surveys. Every patient reported using in a safe place (Table 2). Other strategies endorsed in surveys but not interviews included restricting access to guns and not driving while using/intoxicated. During interviews, patients shared that avoiding use in public when possible was helpful, and one patient stated that using with trusted people allowed for naloxone administration and prevented harms that could be inflicted by others while using (e.g., theft, assault).

Patients shared multiple safer injection practices, including using new needles, not sharing needles, and cleaning the injection site. Another was a tester dose, as described by one patient: “Always doing what we call a tester […] so you do like a 1/10 of what you think you would normally do, just to […] test the strength of it…” (Patient 5).

Other patients who used other routes of administration used a variation of this strategy (i.e., start low and go slow): “I tend to take a little bit at a time and wait to get a reaction […] I might take a couple, I might do one line and just wait […] So, that’s how I try to stay safe [..] by doing it a little at a time” (Patient 6).

Patients reported avoided mixing drugs with alcohol and avoided mixing depressants to reduce risk of overdose in surveys and interviews. Most patients used and recommended buying from a trusted source as a SUS, as shared by one patient: “…always use the same person to get it from […] The same person is very important. It doesn’t matter how much it costs” (Patient 4).

#### Clinician Supports

Clinicians reported discussing some of these with patients, including not using alone and select safer injection practices, though none reported discussing tester shots or safer smoking strategies. Some clinicians offered patients information on syringe service programs. Although few clinicians discussed using safer routes of administration, all clinicians indicated they would be comfortable sharing this strategy when asked directly. Several clinicians described how their team supported patients in finding housing to facilitate using in a safe place. One clinician discussed not using near children when possible to avoid legal consequences, and some discussed not mixing certain drugs (i.e., opioids and benzodiazepines, opioids and alcohol). Few clinicians reported checking whether a patient’s supplier changed.

#### Suggestions/Recommendations

All patients recommended discussing not mixing opioids and stimulants (i.e., speedballing) and safer routes of administration in PC. Most patients suggested discussing not using alone and using in a safe place in PC, and some clinicians expressed desire for patients to have access to safe consumption sites and harm reduction-focused housing. Several clinicians suggested routinely offering safer use supplies, though no clinics currently offered this. Clinicians also desired training on safer smoking practices, as summarized by one clinician: “I’m not sure that I […] have a ton of sort of self-knowledge or resources for [safer smoking strategies]. So I would love to learn more, or have more at hand to be able to counsel patients on” (Clinician 10).

### Ways to Change How Much You Use

#### Patient Use

Patients reported multiple SUS to moderate use in surveys and interviews, including strategies to limit use, discussing withdrawal with clinicians, and MOUD. Patients also endorsed other moderation strategies in surveys, including tolerance breaks (i.e., temporary abstinence to lower tolerance), using after fulfilling obligations, and buying less at a time. In interviews, patients shared specific strategies like only buying after bills were paid, locking up supply to limit access, and only using a certain amount to avoid “nodding.” In interviews and surveys, patients indicated that physician-prescribed MOUD was helpful for reducing or stopping street opioid use. In interviews, one patient reported obtaining MOUD on the street to reduce use of street opioids, reduce risk of contaminated supply, manage chronic pain, and mitigate withdrawal: “The Suboxone, before I ever got on with my doctor’s, I did […] I never wanted to […] use heroin or anything, mainly because […] you never know what dose you’re gonna get. You never know how strong it is […] I would buy [Suboxone] and try to cut them up really small” (Patient 10).

#### Clinician Supports

Only one clinician reported discussing starting low and going slow, especially after a period of abstinence, and few discussed moderation strategies with patients. Most clinicians were very comfortable discussing withdrawal and prescribing MOUD. Clinicians also acknowledged that patients were obtaining MOUD without a prescription as a SUS prior to seeking treatment, as summarized by one clinician: “I have personally never done an induction in the office because 99.9% of these patients that come in have already used Suboxone on the street, have already done an induction, have already gone through withdrawal, and that’s why they’re here” (Clinician 1).

Few clinicians prescribed off-label medications for stimulant use disorder (StUD). Among those who did, mirtazapine, lisdexamfetamine, methylphenidate, and amphetamine/detroxamphetamine were used, though only with a select few patients. As summarized by one clinician: “I mean, it’s literally an *n* of 2. So one has very much reduced his use […] the other one has stopped using IV" (Clinician 9).

#### Suggestions/Recommendations

Most patients recommended all SUS for using less in PC (Table 2). Every patient recommended MOUD, and every clinic offered MOUD. Both patients and clinicians expressed a need for medications for StUD, as summarized by one patient: “I think Suboxone, or Sublocade, is kind of a miracle drug, and I wish they had a drug similar to that for stimulants” (Patient 9).

Other patient recommendations included MOUD scheduling and appointment flexibility and providing information about MOUD treatment on their website. Clinicians echoed the need for appointment flexibility and other system-level changes to improve MOUD treatment, including more time for patient outreach and follow-up.

### General Suggestions/Recommendations

Clinician-reported suggestions that spanned SUS categories included patient assistance with housing, transportation, and employment, home visits, and training in multiple areas (i.e., anti-stigma, compassion, trauma-informed care). Several clinicians acknowledged that patients often know more about SUS, as stated by one clinician: “I think a lot of the patients are pretty savvy about kind of the best ways to prevent [overdose] from happening. I think a lot of them have more knowledge than we do…” (Clinician 6). Clinicians indicated they need more SUS training to better support patient harm reduction goals.

Some clinicians used patient-centered goal-setting and/or motivational interviewing to support SUS use and suggested this be used by all clinicians. When patients were asked what clinicians should know to improve care, most recommendations involved authenticity, active listening, and compassion, as summarized by one patient: “Just be honest and truthful and show that you care” (Patient 6). Supporting patient SUS use also aligned with feedback from another patient, who advised: “Don’t tell them to stop. Obviously, we know what we’re doing. We do know what the effects are and the consequences. I think they should think more about what they can do and be effective” (Patient 4).

## DISCUSSION

We explored the acceptability of offering SUS in PC, SUS used by patients, supports offered by clinicians, and recommendations. Every patient used multiple SUS, and over half recommended every SUS be offered in PC (Table 2). Clinicians provided informational and tangible resources to patients to support SUS use and perceived increasing provision of SUS in PC as acceptable.

Clinicians found prescribing naloxone acceptable, which is consistent with previous work,^25^ and all clinics offered naloxone to patients. However, offering naloxone could be more standardized and offered to all patients who use opioids and/or stimulants, especially in clinics with funding to distribute naloxone on-site without a prescription. Our findings suggest patients might be interested in naloxone for a friend (i.e., third-party prescribing), which is covered by Medicaid in multiple states and increases naloxone dispensing.^40^

Patients reported the SUS of using in a safe place, though this was difficult for patients without housing. Not all patients could use with safe people, but most found spotting (i.e., telling someone when you plan to use) acceptable in the survey. Clinicians could offer in-person (e.g., supervised consumption sites [though only available in the U.S. in New York as of 2024])^41^ and/or remote spotting resources (e.g., Never Use Alone hotline;^42^ in addition to prescribing naloxone.

Many clinicians were interested in offering the same tangible supports as syringe service programs (e.g., safer smoking kits). This echoes results from a recent survey of people who inject drugs^43^ and calls for policy changes in medical settings to allow distribution of safer use supplies.^44,45^ Our findings also align with calls to prescribe syringes in PC to patients who inject drugs,^46,47^ which is legal in approximately half of the U.S.^46^

Patients and clinicians indicated offering fentanyl test strips was acceptable, though neither knew where to access them. Previous research suggests that using fentanyl test strips prompts use of other SUS to reduce risk of overdose when fentanyl is detected, but highlighted the need for multiple SUS to be offered in addition to fentanyl test strips.^48^ A recent pilot study reported positive patient experiences with fentanyl test strip distribution in emergency departments.^49^ Further, evidence suggests that test strips for other drugs (e.g., xylazine, methamphetamine) have adequate sensitivity and are more cost- and time-efficient compared to laboratory-based methods.^50^

Patients and clinicians shared similar views on medication treatments. Both considered medications for opioid use disorder to be life-saving and critical to recovery, and both desired effective medications for stimulant use disorder. However, there were some differences in treatment of physical pain. Although some clinicians cited adequate pain management as a SUS, no patients reported this as a SUS used during interviews. However, multiple patients reported physical pain as a reason for use, suggesting pain management is critical for reducing use of opioids and could be framed as a SUS when discussing treatment options with patients.

SUS are an important component of harm reduction, though not the only aspects that need attention. The National Harm Reduction Coalition defines harm reduction as “a set of practical strategies and ideas aimed at reducing negative consequences associated with drug use,” *and* “a movement for social justice built on a belief in, and respect for, the rights of people who use drugs.”^51^ SUS are practical strategies, but stigma is a common barrier to healthcare,^52^ and many experience discrimination in healthcare settings.^53^ As suggested by clinicians, anti-stigma, trauma-informed care, and compassion training may be needed to address healthcare inequities.

### Strengths, Limitations, and Future Directions

Strengths of this study include a mixed-method design to explore patient SUS perspectives, qualitative data from patients and clinicians, and a diverse patient sample. Although we reached theme-level saturation among patients and clinicians, the sample size was small and may limit generalizability. Our findings may not generalize to PC settings without integrated behavioral health and MOUD programs or those in different geographic locations. We did not assess clinician-perceived acceptability of every SUS, and there are additional SUS not assessed in the patient survey. Patients younger than 30 and clinicians older than 53 were not represented in our sample. It is possible these groups are less comfortable discussing SUS in PC, so more research is needed to assess the acceptability of SUS in PC among younger adult patients and older clinicians. Other limitations include limited clinician racial diversity and lack of SUS for certain routes (e.g., insufflation/snorting, rectal). Clinician recruitment by clinical champions may further limit generalizability. Future directions include identifying SUS for these routes, developing standardized SUS measures, and developing and testing brief SUS interventions.

## CONCLUSION

Patients and clinicians reported offering SUS to patients who use stimulants and/or opioids is an acceptable practice which could improve patient-centered PC and save lives. Patients use multiple SUS to reduce harms and/or use, and clinicians support SUS use through informational and tangible resources. Clinicians desire more SUS training, and brief SUS interventions are needed for PC.

### Researchers’ Positionality Statement

Our team is comprised of researchers and clinicians with diverse gender, sexual orientation, and racial identities. However, our team did not include the voices of Black, Native American/Alaska Native, or Pacific Islander people. Multiple co-authors have lived experience of drug use and SUS use. All team members have the privileged status of receiving higher education. We acknowledge that our identities, experiences, and pro-harm reduction attitudes may have influenced our interpretation of the qualitative data.

## Supplementary Information

Below is the link to the electronic supplementary material.

Supplementary file1 (DOCX 28.9 KB)

## Data Availability

Interview guides are available in Appendices 2 and 3. Quantitative descriptive statistics are available in Tables 1 and 2 and Appendix 1 Tables 3 and 4. Item-level quantitative data and qualitative summaries are available upon request to the corresponding author with completion of a data sharing agreement. Select demographic variables and qualitative transcripts will not be shared to protect participants’ privacy.
