# Supplementary material for: Primary Care Patient and Clinician Perspectives on Safer Use Strategies for Opioids and/or Stimulants: A Mixed-Method Study
Source: J Gen Intern Med. 2025 Mar 4;40(12):2953–61. doi: 10.1007/s11606-025-09418-5 (PMC12463806; doi:10.1007/s11606-025-09418-5)
Supplement: Supplementary file 1 — Supplementary file1 (DOCX 28.9 KB) [file 11606_2025_9418_MOESM1_ESM.docx]

**APPENDIX 1**

*Table 3 Additional Patient Participant Demographics/Social Determinants of Health*

| **Patient demographics/social determinants of health** | ***n* = 9***  *n* (%) |
| --- | --- |
| **City size** |  |
| Small city or town | 1 (11%) |
| Suburb near a large city | 2 (22%) |
| Large city | 6 (67%) |
| **Education** |  |
| Some high school, no diploma | 2 (22%) |
| High school or equivalent | 2 (22%) |
| Some college, no degree | 3 (33%) |
| Trade/technical/vocational training | 1 (11%) |
| Bachelor’s degree | 1 (11%) |
| **Employment status** |  |
| Full time paid work | 3 (33%) |
| Part time paid work | 2 (22%) |
| Receiving disability benefits | 2 (22%) |
| Receiving disability benefits and part time paid work | 1 (11%) |
| Unemployed | 1 (11%) |
| **Disability status** |  |
| Yes (identifies as disabled) | 7 (78%) |
| **Housing status** |  |
| A house or apartment you own or rent | 7 (78%) |
| Someone else’s house or apartment | 1 (11%) |
| A shelter or transitional housing facility | 1 (11%) |
| **Financial comfort** |  |
| Can’t make ends meet | 2 (22%) |
| Have just enough to get by | 4 (44%) |
| Are comfortable | 2 (22%) |
| More than comfortable | 1 (11%) |

*Note:* Percentages reflect endorsements from available data.

*One patient did not complete demographic survey (patient *N* = 10).

Table 4

*Patient Drug Use Experience*

| **Drug use experience** | **Past-month use**  (*n* = 9)* | | **Lifetime use**  (*n* = 9)* | |  |
| --- | --- | --- | --- | --- | --- |
| Alcohol | 4 (44%) | | 9 (100%) | |  |
| Cannabis | 4 (44%) | | 8 (89%) | |  |
| Cocaine | 3 (33%) | | 8 (89%) | |  |
| Methamphetamine | 2 (22%) | | 6 (67%) | |  |
| Stimulant medications NOT prescribed for you | 0 (0%) | | 4 (44%) | |  |
| Stimulant medication prescribed to you at higher doses or more often than prescribed | 0 (0%) | | 1 (11%) | |  |
| Opioid medications prescribed for you at higher doses or  more often than prescribed | 0 (0%) | | 3 (33%) | |  |
| Opioid medication NOT prescribed for you (not  including fentanyl) | 0 (0%) | | 5 (56%) | |  |
| Heroin | 0 (0%) | | 6 (67%) | |  |
| Fentanyl | 1 (11%) | | 5 (56%) | |  |
| Hallucinogens (LSD, mushrooms) | 0 (0%) | | 5 (56%) | |  |
| More than 1 substance in a day | 5 (56%) | | 9 (100%) | |  |
| **Route(s) of administration** | Stimulants  (*n* = 8) | Opioids (excluding fentanyl)  (*n* = 6) | | Fentanyl  (*n* = 5) | |
| Swallow pills | 4 (50%) | 2 (33%) | | 0 (0%) | |
| Smoking | 7 (88%) | 3 (50%) | | 2 (40%) | |
| Snorting | 4 (50%) | 3 (50%) | | 1 (20%) | |
| Injecting | 5 (63%) | 6 (100%) | | 1 (20%) | |
| Rectal (boofing/shelving) | 1 (13%) | 0 (0%) | | 0 (0%) | |
| Other (please specify) | 0 (0%) | 0 (0%) | | 1 (20%) | |

*Note.* Percentages reflect endorsements from available data. Fentanyl was asked separately from opioid questions to collect data on fentanyl-related experiences. Participants were only asked for drug-specific route of administration if reporting any use of particular drug class. Participants could select all routes of administration used. Percentages will not equal 100%.

*One patient did not complete survey (patient *N* = 10).

^†^Other route of administration used for fentanyl was taking transdermal patch sublingually.

**APPENDIX 2**

*Relevant Prompts from Patient Semi-Structured Interview Guide*

**Have you ever heard the term “harm reduction”? What does that mean to you?**

**(Affirm response or clarify)**

**Because harm reduction can mean so many things, we’d like to share the definition we use:** “Harm reduction is a set of practical strategies and ideas aimed at reducing negative consequences associated with drug use. Harm reduction is also a movement for social justice built on a belief in, and respect for, the rights of people who use drugs.”

**What do you think about primary care offering harm reduction strategies* to patients?**

- *Ex: naloxone/Narcan, fentanyl test strips, safer use supplies, safer use counseling

**What do you think about being offered fentanyl testing strips in your primary care clinic?**

- **Would you take some for yourself?**
- **Would you take some for friends/others?**
- **Where could you get strips if you wanted them?**

**What do you think about talking to a provider about ways to safer while using?**

**Do you think other patients who use drugs might be interested in something like this?**

- **Why/why not?**

**What do you think would make people feel safer to discuss drug use in primary care?**

- **What should they *not* do?**

**Thank you. Now, I’d like to learn more about your experience with drugs and different services if that’s okay. Please remember you can refuse to answer any question at any time.**

**What were/are the reasons you use(d) drugs?**

- Perceived benefits
- Ex: Coping with MH, trauma, living situation, pain, sleep

**What kinds of things have you done to stay safe and/or avoid consequences while using drugs?**

- Ex: not use alone, use a little at a time, don’t mix certain drugs, don’t drive, use in a safe place, buy less, drink water

**What kinds of specific consequences do you try to avoid while using drugs?**

- Ex: overdose, withdrawal, legal, injury, infectious disease, social problems

**When using, how often do you use alone?**

- **Who would you be comfortable using around?**

**What (if anything) has been helpful for you to stay safe and/or use less?**

- Ex: naloxone/Narcan, community programs/groups, NA/AA, syringe service programs, safer use supplies, medication, social services (case management)

**Anything else you think primary care teams and researchers should know to improve their work with people who use drugs and/or alcohol?**

**APPENDIX 3**

*Relevant Prompts from Clinician Semi-Structured Interview Guide*

**Which drugs are commonly used by patients at your clinic?**

**What kinds of problems do patients who use opioids and/or stimulants typically present with?**

- **What are the biggest health concerns for these patients?**
  - Drug toxicity/overdose concerns?

**When thinking about patients who use drugs, what are your biggest priorities/concerns?**

- **Which drug-related consequences are a priority for you/your clinic?**

**How important is increasing provision of harm reduction tools and education to you?**

- **Why?**
- **How important is it to others in the clinic?**
  - **Leadership?**
  - **Patients?**

**Which drugs do you think should prioritized when developing a harm reduction intervention for patients?**

- What about nonmedical opioids and/or stimulants use? Why or why not?

**What kinds of behavioral strategies (not meds or naloxone) do you discuss with patients?**

- Ex: not using alone, eating/drinking before/during use

**What kinds of harm reduction strategies are you comfortable offering to patients?**

- **What about safer injection practices? Safer smoking practices?**
- **Fentanyl test strips?**

**What is currently offered to patients who use drugs nonmedically and/or alcohol?**

- Ex: MOUD, SBIRT, HR counseling, ID screening/testing/treatment, naloxone, wound care, mental health care

**What works well?**

**What needs improvement?**

**What else do you think could feasibly be offered to patients who use drugs nonmedically in your clinic?**

**If you could make one change to improve care for patients who use drugs, what would it be?**
